# Gradient Relationship between Increased Mean Corpuscular Volume and Mortality Associated with Cerebral Ischemic Stroke and Ischemic Heart Disease: A Longitudinal Study on 66,294 Taiwanese

**DOI:** 10.1038/s41598-018-34403-w

**Published:** 2018-11-08

**Authors:** Tzy-Haw Wu, Jean Ching-Yuan Fann, Sam Li-Sheng Chen, Amy Ming-Fang Yen, Chiung-Jung Wen, Yun-Ru Lu, Hsiu-Hsi Chen, Sherry Yueh-Hsia Chiu, Horng-Huei Liou

**Affiliations:** 1 Department of Internal Medicine, National Taiwan University Hospital, Taipei, Taiwan; 2 Department of Health Industry Management, College of Healthcare Management, Kainan University, Taoyuan, Taiwan; 3 School of Oral Hygiene, College of Oral Medicine, Taipei Medical University, Taipei, Taiwan; 4 Department of Geriatrics and Gerontology, National Taiwan University Hospital, Taipei, Taiwan; 5 Department of Family Medicine, National Taiwan University Hospital, Taipei, Taiwan; 6 Department of Neurology, China Medical University Hospital, Taipei, Taiwan; 7 Graduate Institute of Epidemiology and Preventive Medicine, College of Public Health, National Taiwan University, Taipei, Taiwan; 8 Department of Health Care Management and Healthy Aging Research Center, Chang Gung University, Taoyuan, Taiwan; 9 Division of Hepatogastroenterology, Department of Internal Medicine, Kaohsiung Chang Gung Memorial Hospital, Kaohsiung, Taiwan; 10 Department of Neurology and Pharmacology, National Taiwan University Hospital and College of Medicine, National Taiwan University, Taipei, Taiwan

## Abstract

The gradient relationship between mean corpuscular volume (MCV) and mortality due to ischemic vascular disease has not been researched using a large-scale population-based study. This study evaluated the association between MCV and death attributable to cerebrovascular disease (CVD) and cardiovascular disease (CAD) in a large population- and community-based Taiwanese cohort. A longitudinal study with a 9-year follow-up was conducted to evaluate individuals aged 20 years or older who had participated in the Keelung (the northernmost city in Taiwan) community-based integrated screening (abbreviated as KCIS) program since September 1999. The mortality rates associated with CVD and CAD were classified across a range of different MCV levels. Increased MCV levels were associated with an increased risk of CVD/CAD-related death (adjusted hazard ratio [aHR] = 1.42, trend test P = 0.0119). Marginally statistically significant associations were noted for specific deaths from ischemic heart disease (aHR = 1.44, trend test P = 0.0992) and cerebral ischemic stroke (aHR = 1.66, trend test P = 0.0667), respectively, but no significant gradient relationship was noted for death from cerebral hemorrhage stroke (aHR = 1.23, trend test, P = 0.6278). A gradient relationship between baseline MCV level and CVD/CAD-related death was noted, but whether such gradient relationships existed for two specific deaths and how these relationships may be confounded by extraneous factors that were not considered here should be investigated in the future.

## Introduction

In light of the global burden of disease (GBD) based on the World Health Organization (WHO) database, ischemic heart disease (death: 14.8%, disability: 6.1%) and stroke (death: 11.8%, disability: 4.5%) are the second and third most common causes of death and disability. These diseases are independent of gender and the country’s economic status (developing or developed), which has led to a dramatically increased absolute number of patients on healthcare encumbrance^1^. Effective preventive strategies for ischemic disease and stroke in Asia are important for stopping the increasing trend, especially given that more than 60% of the world’s population is located in Asia^2^. Therefore, the importance of identifying the potential risk factors that are responsible for the occurrence of stroke cannot be overemphasized. Many major risk factors, such as hypertension, diabetes mellitus, hyperlipidemia, and heart disease, can lead to stroke^1–5^. However, some risk factors, particularly those related to hemorheology, have not been well defined. Platelets play an important role in stroke, and antiplatelet agents are one of the main interventions for the prevention of primary and secondary ischemic stroke (IS)^6,7^. Several studies have revealed the relationship between stroke and white blood cell (WBC) count and subtype, and the results have demonstrated that WBC count is an independent predictor of stroke severity and outcome after acute IS^8^. Another study found that an elevated WBC count was positively associated with poor clinical outcomes in patients with acute IS^9^.

Despite these findings, how red blood cells affect stroke risk has not been fully elucidated. A previous study explored the association between red blood cell distribution width (RDW)^10^ and susceptibility to cardiovascular events^11^. Another study addressed the relationship between RDW and the incidence of cardiovascular events in people with coronary disease^12^. Other studies have examined RDW in relation to the risk of cardiovascular morbidity and mortality^13,14^. RDW is an indicator of variation in red blood cell (RBC) size within blood samples, not the actual width of individual RBCs. RDW-coefficient of variation (CV), a measure of RDW, is derived from the equation (1 unit of standard deviation [SD] of erythrocyte volumes divided by mean corpuscular volume [MCV]) × 100%^15^. Therefore, a relationship exists between RDW and MCV. MCV is the common item of the complete blood count (CBC) examination package without any transformation. However, few studies have investigated the association of cerebrovascular disease (CVD)/cardiovascular disease (CAD). One case-control study conducted in 2001 identified an association between MCV and peripheral arterial disease in male subjects^16^. MCV may also predict left atrial stasis in patients with nonvalvular atrial fibrillation^17^. Based on these findings, elevated MCV may play a significant role in ischemic vascular disease. However, no study has evaluated the temporal relationship between MCV and stroke in a large population- and community-based longitudinal study. In the present study, empirical data from the Keelung Community-based Integrated Screening (KCIS) program in Taiwan were evaluated. The KCIS program collected information on factors associated with metabolic syndrome and biological markers (including MCV). In this large longitudinal study, the relationship between MCV and mortality from various types of cardiovascular disease, in addition to other established factors, was evaluated during a long-term follow-up.

## Results

After a 9-year follow-up (median time, 6.21 years) period, CVD- and CAD-specific mortality rates were estimated based on demographics, biomarkers, and lifestyle factors (Table 1). In the univariate analysis, the risk of CVD/CAD mortality was associated with age, male gender, lower education, abnormal metabolic syndrome factors, elevated WBC, and anemia. In addition to these well-established risk factors, the mortality rate of CVD/CAD according to MCV level was as follows: 3.99 per 100,000 (95% CI: 3.29, 4.69) at an MCV ≥99 fL compared with 1.44 per 100,000 (95% CI: 1.00–1.88) at an MCV <80 fL. Using the cutoff for anemia, patients with anemia showed an increased risk of CAD/CVD mortality compared to those without anemia (4.11 vs. 1.48 per 100,000). Those who had an aspartate aminotransferase-to-alanine aminotransferase ratio (AST/ALT ratio) greater than 1, an estimated glomerular filtration rate (eGFR) less than 60, and infrequent fruit intake had increased CAD/CVD mortality compared with each reference group. The specific mortality rates for CVD/CAD based on the associated factors are listed in Table 1.

**Table 1:** Mortality rates due to CVA/CAD based on different physiological and biochemical factors.

Variable	Class	Overall subjects	Follow-up person-years	CVD/CAD death	CVD/CAD mortality rate (95% CI)
Age	40–49	25370	144032.32	28	0.19 (0.12–0.26)
	50–59	18520	107126.29	53	0.49 (0.36–0.62)
	60–69	12486	76490.50	122	1.59 (1.31–1.87)
	70+	9918	56695.52	460	8.11 (7.37–8.85)
Gender	Female	39908	234872.05	264	1.12 (0.98–1.26)
	Male	26386	149472.57	399	2.67 (2.41–2.93)
Education (years)	>12	9914	52673.29	28	0.53 (0.33–0.73)
	6–12	29301	166020.66	153	0.92 (0.77–1.07)
	<6	27052	165432.66	482	2.91 (2.65–3.17)
Waist	Normal	42001	243448.45	325	1.33 (1.18–1.48)
	Abnormal	24293	140896.17	338	2.40 (2.14–2.66)
Triglycerides	Normal	45745	265097.07	409	1.54 (1.39–1.69)
	Abnormal	20549	119247.55	254	2.13 (1.87–2.39)
HDL	Normal	48656	282018.15	425	1.51 (1.37–1.65)
	Abnormal	17638	102326.47	238	2.33 (2.03–2.63)
Blood pressure	Normal	30768	172997.02	147	0.85 (0.71–0.99)
	Abnormal	35526	211347.60	516	2.44 (2.23–2.65)
FPG	Normal	47538	274990.30	341	1.24 (1.11–1.37)
	Abnormal	18756	109354.32	322	2.94 (2.62–3.26)
Cigarette smoking	None	48683	286200.13	432	1.51 (1.37–1.65)
	Quit	4438	24334.34	56	2.30 (1.70–2.90)
	Current	13129	73518.96	175	2.38 (2.03–2.73)
Alcohol consumption	None	50814	295768.20	501	1.69 (1.54–1.84)
	Quit	2494	14179.13	49	3.46 (2.49–4.43)
	Current	12710	72515.53	111	1.53 (1.25–1.81)
Exercise	Infrequent	22993	132911.00	254	1.91 (1.67–2.15)
	Frequent	41990	243249.93	390	1.60 (1.44–1.76)
WBC	<6.2 × 10^3^	31867	188966.54	256	1.35 (1.18–1.52)
	≥6.2 × 10^3^	34426	195372.87	407	2.08 (1.88–2.28)
MCV level (fL)	<80	4988	28387.16	41	1.44 (1.00–1.88)
	80–94.9	41851	239451.30	334	1.39 (1.24–1.54)
	95–98.9	14212	85457.43	164	1.92 (1.63–2.21)
	≥99	5243	31048.72	124	3.99 (3.29–4.69)
Anemia	No	59859	348807.40	517	1.48 (1.35–1.61)
	Yes	6435	35537.22	146	4.11 (3.44–4.78)
AST/ALT ratio	≤1	28950	167390.16	194	1.16 (1.00–1.32)
	>1	37340	216923.64	468	2.16 (1.96–2.36)
eGFR	≥60	59134	343578.35	336	0.98 (0.88–1.08)
	<60	7159	40758.93	327	8.02 (7.15–8.89)
Seafood intake (times/week)	0–2	30949	174684.94	329	1.89 (1.68–2.08)
	≥3	32994	195053.58	306	1.57 (1.39–1.75)
Bean intake (times/week)	0–2	31975	186638.53	371	1.99 (1.79–2.19)
	≥3	32031	183571.68	262	1.43 (1.26–1.60)
Milk intake (times/week)	0–2	33870	191331.80	322	1.68 (1.50–1.86)
	≥3	30055	178376.47	316	1.77 (1.57–1.97)
Meat intake (pieces/day)	0–1	48976	283975.66	508	1.79 (1.63–1.95)
	≥2	14872	85304.57	128	1.50 (1.24–1.76)
Fruit intake (times/week)	0–2	14100	76621.38	195	2.54 (2.18–2.90)
	≥3	49081	287089.58	425	1.48 (1.34–1.62)
Vegetable intake (bowls/day)	0–1	45908	264130.89	480	1.82 (1.66–1.98)
	≥2	18322	107943.07	158	1.46 (1.23–1.69)
Exercise	Nonregular	22993	132911.00	254	1.91 (1.67–2.15)
	Regular	41990	243249.93	390	1.60 (1.44–1.76)

MCV: Mean corpuscular volume.

FPG: fasting plasma glucose; Anemia: males, hemoglobin <13; females, hemoglobin <12; Nonanemic: males, hemoglobin ≥13; females, hemoglobin ≥12.

The distributions of baseline MCV levels were as follows: 70.7%, 21.4%, and 7.9% for MCV levels of <95, 95–98.9, and ≥99 fL, respectively. In total, 3642 deaths occurred among 66,294 subjects over a median follow-up of 6.21 years. A greater proportion of participants who died had an elevated MCV level (17.7%) compared with the proportion of individuals who were still alive (7.3%). Of the 3642 total deaths, there were 663 deaths from CVD/CAD, and this group included a slightly increased proportion of participants with an elevated MCV level. A greater proportion of participants who had specific causes of death related to CVD/CAD, particularly cerebral IS and ischemic heart disease (IHD), demonstrated elevated MCV levels (Table 2).

**Table 2:** Causes of death according to MCV level.

Type	Classification	MCV levels						Total N
		<95 (fL)		95–98.9 (fL)		≥99 (fL)		
		N	(%)	N	(%)	N	(%)	
All subjects		46839	(70.7)	14212	(21.4)	5243	(7.9)	66294
Overall Status	Alive	44773	(71.5)	13280	(21.2)	4599	(7.3)	62652
	Dead	2066	(56.7)	932	(25.6)	644	(17.7)	3642
Cause of death	CVD/CAD-related death	375	(56.6)	164	(24.7)	124	(18.7)	663
	Cancer-related death	679	(56.5)	317	(26.4)	206	(17.1)	1202
	Other death	1012	(56.9)	451	(25.4)	314	(17.7)	1777
CVD/CAD -related death*	Cerebral ischemic stroke death	48	(51.1)	28	(29.8)	18	(19.1)	94
	Cerebral hemorrhagic stroke death	62	(62.6)	23	(23.2)	14	(14.2)	99
	Hypertensive disease death	30	(56.6)	13	(24.5)	10	(18.9)	53
	Ischemic heart disease	154	(56.4)	66	(24.2)	53	(19.4)	273
	Heart failure death	38	(60.3)	15	(23.8)	10	(15.9)	63
	Pulmonary heart/circulation death	0	(0.0)	2	(66.7)	1	(33.3)	3
	Arteries, arterioles, capillaries death	9	(60.0)	4	(26.7)	2	(13.3)	15
	Late effect of cerebrovascular dis. death	34	(54.0)	13	(20.6)	16	(25.4)	63

MCV: Mean corpuscular volume.

*Please see supplemental document as classification for CVD/CAD-related death.

**Figure 1:**
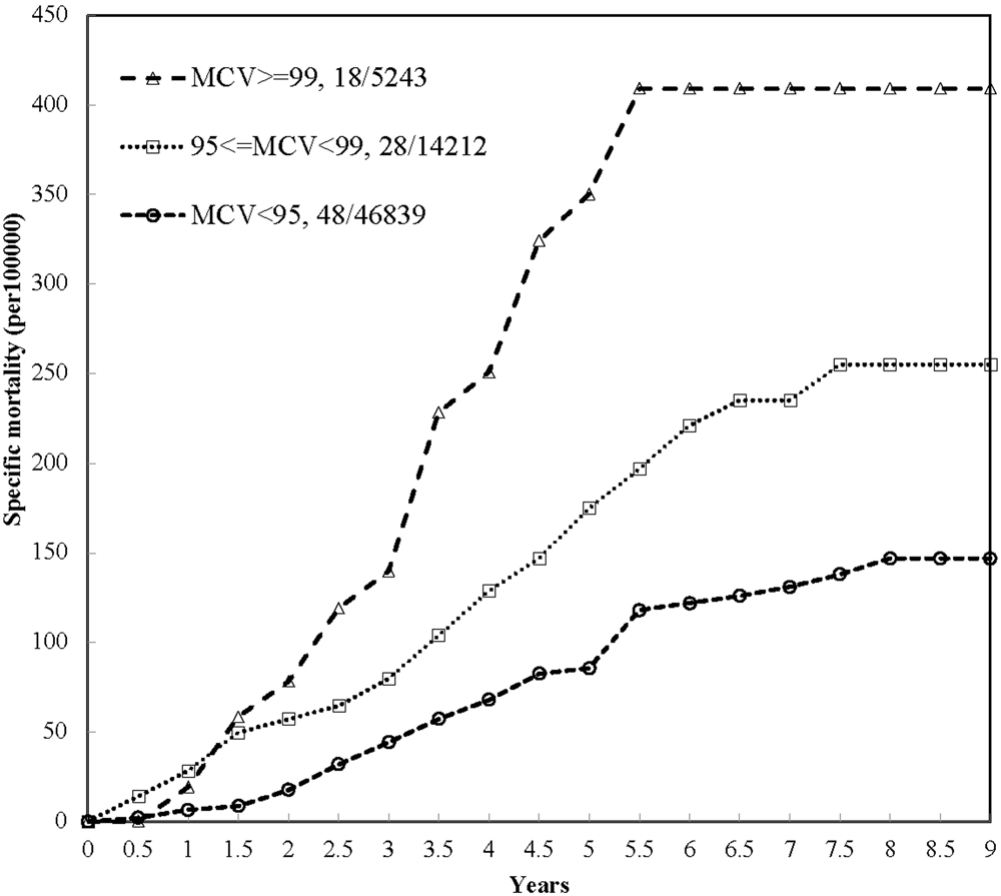
Cumulative mortality of cerebral ischemic stroke causes based on MCV level.

Based on these data, assessing the MCV level and the risk of death with a Cox proportional hazards regression model fitted with the proportional hazards assumption, the time-dependent variable showed a lack of statistical significance on interaction (p-value = 0.6731, see Supplemental Fig. 2). To examine the association between baseline MCV level and specific causes of death related to CVD/CAD, individuals with an elevated MCV level were compared to those with a low MCV level using univariate analysis. The results showed a significantly increased risk for those with an elevated MCV level (hazard ratio [HR] = 2.84; 95% confidence interval [CI]: 2.32–3.48). The crude HRs for all variables are reported in Supplemental Table 1. Additional factors, including lower education level, components of metabolic syndrome, cigarette smoking, elevated WBC and anemia, were also significant. After adjusting for these factors, an elevated MCV level still led to a significantly increased risk of death related to CVD/CAD (aHR = 1.42; 95% CI: 1.15–1.76). There was also a significant gradient relationship between MCV level and risk of death (p-value for trend test = 0.0119) (Table 3). The cumulative specific mortality rate for CVD/CAD was used to compare the risk corresponding to different MCV levels. The highest cumulative specific mortality rate for CVD/CAD was found in subjects with an MCV ≥99 fL (Fig. 1).

**Table 3:** Adjusted hazard ratios for risk of specific causes of mortality.

Variable	Classification	CVD/CAD-related death	Cerebral ischemic stroke death	Cerebral hemorrhagic stroke death	Ischemic heart disease death
		aHR (95% CI)	aHR (95% CI)	aHR (95% CI)	aHR (95% CI)
MCV level	95–98.9 vs. <95	0.96 (0.80–1.16)	1.30 (0.81–2.08)	0.96 (0.59–1.56)	0.93 (0.69–1.26)
	≥99 vs. <95	1.42 (1.15–1.76)	1.66 (0.96–2.88)	1.23 (0.68–2.23)	1.44 (1.04–2.00)
Trend test (p-value)		0.0119	0.0667	0.6278	0.0992
Age		1.10 (1.10–1.11)	1.07 (1.05–1.10)	1.06 (1.04–1.09)	1.11 (1.10–1.13)
Gender	Male vs. female	1.74 (1.44–2.12)	1.88 (1.22–2.89)	1.34 (0.88–2.03)	1.83 (1.37–2.46)
Years of	6–12 vs. >12 yr	1.60 (1.05–2.42)	5.08 (0.68–38.23)	—	—
education	<6 vs. >12 yr	1.77 (1.18–2.66)	9.42 (1.30–68.38)	—	—
HDL	Abnormal vs. normal	1.31 (1.11–1.55)	—	—	1.50 (1.16–1.95)
Blood pressure	Abnormal vs. normal	1.41 (1.16–1.70)	1.61 (0.95–2.72)	1.99 (1.19–3.35)	—
FPG	Abnormal vs. normal	1.37 (1.16–1.60)	1.70 (1.12–2.58)	—	1.32 (1.03–1.70)
Cigarette smoking	Quit vs. none	1.05 (0.76–1.45)	—	—	0.86 (0.50–1.47)
	Current vs. none	1.27 (1.03–1.57)	—	—	1.41 (1.02–1.93)
Alcohol drinking	Quit vs. none	1.55 (1.12–2.14)	—	—	1.67 (1.02–2.73)
	Current vs. none	0.95 (0.75–1.21)	—	—	0.84 (0.57–1.22)
Anemia	Yes vs. no	1.83 (1.50–2.24)	1.62 (0.96–2.74)	2.13 (1.28–3.55)	1.86 (1.37–2.53)
WBC	≥6.2 vs. <6.2	1.34 (1.14–1.58)	1.29 (0.85–1.97)	1.27 (0.84–1.91)	1.37 (1.06–1.77)
AST/ALT ratio	>1 vs. ≤1	1.23 (1.03–1.48)	1.62 (0.99–2.65)	1.29 (0.81–2.05)	1.12 (0.85–1.49)
eGFR	<60 vs. ≥60	1.67 (1.40–2.00)	2.18 (1.37–3.48)	1.71 (1.05–2.78)	1.47 (1.11–1.94)
Exercise	Regular vs. Nonregular	0.73 (0.62–0.86)	—	0.62 (0.41–0.93)	0.76 (0.59–0.99)
Fruit intake	≥3 vs. 0–2 times/week	0.74 (0.62–0.89)	—	—	0.64 (0.49–0.84)

MCV: Mean corpuscular volume

FPG: fasting plasma glucose; Anemia: males, hemoglobin <13; females, hemoglobin <12.

To determine the association between baseline MCV level and specific mortality risk due to IS or cerebral hemorrhagic stroke (CHS), outcomes were stratified by cause of death. Conventional risk factors, including components of metabolic syndrome, hypertension, hyperglycemia, and anemia, had significant effects on IS-related death. Additionally, an elevated MCV level was a significant risk factor compared to a low MCV level (HR = 3.24; 95% CI: 1.88–5.56). After adjusting for other significant factors, an elevated MCV level showed a marginally significant association with IS-related death (aHR = 1.66; 95% CI: 0.96–2.88), and the trend was of borderline statistical significance (p-value = 0.067).

In addition to the above well-known factors, an elevated MCV level was also statistically significantly associated with an increased risk of CHS-related death (HR = 1.95; 95% CI: 1.09–3.49); however, an elevated MCV level showed a lack of a significant effect on CHS-related death (aHR = 1.23; 95% CI: 0.68–2.23) after adjusting for other factors. In the univariate analysis, the effect of MCV on specific mortality from IHD was statistically significant (HR = 2.98; 95% CI: 2.18–4.07). After adjusting for potential confounding factors, an elevated MCV level remained significantly associated with the risk of death from IHD (aHR = 1.44; 95% CI: 1.04–2.00), but the gradient relationship between MCV level and IHD-related death was marginally statistically significant (p-value for trend test = 0.0992) (Table 3). Excluding subjects who had MCV <60 fL from additional analyses, the results did not significantly change (data not shown). Using a continuous level of hemoglobin (Hb) in the multivariable analysis instead of anemia status, the association between MCV level and risk for CVD/CAD-related death did not substantially change, but the association with IS-related death was attenuated (see Supplement Table 3).

To further assess the effect of change in MCV on CVD and related mortality, an analysis based on the 34,537 subjects who attended two or more screenings was conducted. When the difference in MCV was examined as a continuous variable, there was no significant increase in CVD/CAD-, IS-, CHS-, or IHD-related mortality in either the univariate or multivariable analysis. When the difference in MCV was assessed as a dichotomous categorical variable, the crude difference above −0.5 (overall mean difference in MCV) was associated with a significant increase in CVD/CAD-related mortality. Participants with a level above the mean difference had 1.96 times the risk of CHS-related mortality compared with those with levels below the mean difference. However, there was a lack of significant findings among the causes of mortality after adjusting for age, gender, baseline MCV, and anemia. The influence of the rate of change in MCV on mortality was evaluated using the difference in MCV divided by the time between two MCV measurements. The results show that the change rate far above the mean value (−0.13) led to a 1.99-fold greater risk of CHD-related mortality compared to that below the mean value, but this relationship was not statistically significant in the multivariable analysis after adjustment for age, gender, baseline MCV, and anemia. The lack of significance may be due to the adjustment for baseline MCV in the multivariable analysis (Table 4).

**Table 4:** Effect of MCV change on the risk of specific causes of mortality.

Variable	Model	CVD/CAD-related death	Cerebral ischemic stroke death	Cerebral hemorrhagic stroke death	Ischemic heart disease death
		aHR (95% CI)	aHR (95% CI)	aHR (95% CI)	aHR (95% CI)
Difference in MCV (continuous)	Univariate	1.02 (0.99–1.06)	1.03 (0.93–1.15)	1.06 (0.99–1.14)	0.99 (0.94–1.05)
	Multivariable*	1.01 (0.97–1.05)	1.06 (0.93–1.20)	1.05 (0.97–1.14)	0.98 (0.92–1.05)
Difference in MCV ≥−0.5 vs. <−0.5	Univariate	1.36 (1.04–1.77)	1.58 (0.68–3.65)	1.96 (1.03–3.74)	1.24 (0.82–1.86)
	Multivariable*	1.18 (0.90–1.54)	1.56 (0.66–3.66)	1.69 (0.88–3.25)	1.12 (0.74–1.71)
Change rate in MCV^#^ (continuous)	Univariate	1.03 (0.94–1.13)	1.02 (0.77–1.36)	1.09 (0.97–1.22)	1.02 (0.88–1.19)
	Multivariable*	1.00 (0.91–1.10)	1.05 (0.77–1.42)	1.08 (0.91–1.28)	1.01 (0.87–1.18)
Change rate in MCV^#^ ≥−0.13 vs. <−0.13	Univariate	1.37 (1.06–1.79)	1.60 (0.69–3.70)	1.99 (1.04–3.79)	1.25 (0.83–1.89)
	Multivariable*	1.19 (0.91–1.55)	1.58 (0.67–3.71)	1.71 (0.89–3.29)	1.13 (0.75–1.72)

MCV: Mean corpuscular volume.

*Multivariable analysis adjusted for age, gender, and baseline MCV, and anemia.

^#^Change rate in MCV = difference in MCV/duration in years.

## Discussion

In this study, 9-year follow-up data from a community-based, integrated screening program were used to identify the gradient relationship between MCV level and stroke mortality, which has not been previously studied in a large population-based cohort study. An elevated MCV level (≥99 fL) was an independent risk factor for CVD- and CAD-related death, with a possible larger impact on death caused by IS and IHD but less influence on CHS. These findings may suggest that the mechanisms of IS and CHS differ. The results remained significant after adjusting for multiple CVD and CAD risk factors. Overall, an elevated MCV level (>99 fL) was associated with a 42% increase in the risk of death related to CVA/CAD and 66% and 44% increases in the risk of death related to IS and IHD, respectively, compared with a low MCV level (<95 fL). Importantly, these findings were further confirmed by the significance of the dose-response relationship between MCV level and risk of death. According to the annual hazard rate of mortality due to cerebral ischemia (CIS), shown in Supplemental Fig. 1, subjects with a MCV ≥99 were at a significantly increased risk from the beginning of the study until the 2-year follow-up. After this, the risk gradually declined until the 5-year follow-up and then plateaued after 6 years of follow-up (Fig. 1). This finding indicates that individuals aged 40 and older with an elevated MCV level should be closely monitored for five years to reduce the risk of death due to ischemic disease.

The novel findings mentioned above are in contrast to the results of most previous studies in several aspects. First, the target populations in the majority of these previous studies were based on consecutive clinical series, whereas the present study was based on a community-based screening program representative of the general population.

Second, most studies have focused on RDW rather than MCV. A randomized trial examined the relationship between RDW and cardiovascular event rate in people with coronary disease and concluded that a greater RDW was a risk factor for death and cardiovascular events^11^. Another community cohort study examining RDW and the risk of cardiovascular events and mortality found that an elevated RDW was associated with an increased risk of mortality, but not the development of CAD, in the general population^11^. A large population-based cohort study from the healthcare maintenance organization in Israel reported that the risk of cardiovascular morbidity and all-cause mortality was significantly associated with an increase in the percentage of RDW^13^. A Swedish cohort conducted by Malmo revealed that the incidence of fatal coronary disease increased with increasing RDW, but this relationship was not observed for nonfatal disease^14^. Collectively, these studies reveal that elevated RDW is associated with increased cardiovascular events and deaths, but it should be noted that MCV was not evaluated. A previous case-control study found an association between MCV and peripheral arterial disease in males and concluded that an elevated MCV is a predictor of symptomatic peripheral arterial disease^15^; however, the influence of MCV on the arteries in the brain and heart was not assessed. A previous study using MCV and RDW to predict left atrial stasis in patients with non-valvular atrial fibrillation^17^ revealed that both parameters may be markers of left atrial stasis. However, no studies have focused on the relationship between MCV and CVD/CAD mortality, especially in IS and IHD. The third unique feature of this study is that the outcomes included not only all-cause mortality but also various causes of death. Finally, the temporal relationship between MCV and CVD/CAD mortality was further reinforced by a gradient relationship.

There are several reasons for the biological plausibility of MCV. First, the finding that elevated MCV increased the mortality associated with CVD and CAD can be explained by the pathophysiology of cardiovascular and cerebrovascular ischemia. According to the general role of cardio-cerebral blood rheology, cardio-cerebral blood flow is reduced by increased blood viscosity^18^. Factors that affect blood viscosity include hematocrit, erythrocyte aggregation, erythrocyte flexibility^19,20^, platelet aggregation and plasma viscosity^19^. Hematocrit is a major factor that influences blood viscosity, and there is a logarithmic relationship between viscosity and hematocrit^19^. Hematocrit plays an important role in blood viscosity, which may also contribute to cerebral and heart ischemia. However, as such information on hematocrit examination was not available in the present cohort, its independent role in the mortality of cerebral ischemic stroke and IHD could not be assessed. However, such an interesting subject is worthy further investigation.

Second, anemia and hemoglobin levels are strong predictors of the development of CVD- and CAD-related mortality in various populations^21–24^. Previous studies have revealed the significant effect of hemoglobin on CVD and CAD. Undoubtedly, when investigating the risk factors for CVD and CAD, hemoglobin should be considered. Because the current study data were based on an integrated screening that included various biomarkers and general CBCs, these markers could be adjusted for in the multivariable analysis. The results showed that hemoglobin concentration has a significant effect on both CVD and CAD, similar to previous findings.

Third, in addition to gender and age, MCV level is associated with nutritional deficiency, such as deficiency in vitamin B12, iron, or folic acid^25^, and some studies have also revealed the significant impact of deficiencies in vitamin B12 and folate on IS^26^. The putative mechanism that drives an increase in MCV level might result from these deficiencies, which implies that MCV may be a risk marker rather than a risk factor for CVD/CAD-related death. Moreover, factors affecting MCV also include existing diseases (e.g., abnormal liver function)^25,27,28^ and lifestyle factors (e.g., alcohol consumption, cigarette smoking, and vegetable intake)^29^. The association between MCV level and CVD/CAD-related death may be partially explained by these factors. In the multivariable regression analysis, the statistically significant gradient relationship with an emphasis on IS- and CHD-related deaths was attenuated to borderline statistical significance when these additional variables, such as abnormal liver function, eGFR, exercise, and vegetable intake, were controlled in the model. Significant gradient relationships were shown after removing these variables possibly associated with MCV level, as noted in Supplementary Table 2. The findings should therefore be interpreted with great caution because the statistical power may not be sufficient when more variables are adjusted for.

This study has several potential limitations. First, because the screening procedure did not include RDW data, MCV could not be compared with RDW in this cohort study. According to other study results, RDW may also be a risk factor for CAD^11,12^. To identify the best marker, RDW should be evaluated in a future study. Second, no information on IS subtypes, such as large-vessel IS, small-vessel IS or cardiogenic stroke, was available, and therefore, the relationships between stroke subtype and MCV level could not be specifically assessed. Third, no detailed information on anemia subtypes (e.g., iron-deficiency anemia, β-thalassemia, and sickle cell anemia) was available, which prevented further analysis of this variable despite the fact that different types of anemia cause different vascular injuries^21–24^. Several other factors affecting MCV were not considered in the current study, including other diseases (e.g., hypothyroidism, metabolic disorder, rheumatoid arthritis, and cancer)^25,27,28^ and drug use (e.g., allopurinol and phenytoin for modulating purine metabolism, hydroxyurea and trimethoprim for inhibiting pyrimidine synthesis, or metformin for decreasing vitamin B12 absorption)^30^. Third, the sparsity of deaths may limit the statistically significant associations, particularly when two specific deaths were investigated. A larger study is therefore required to gain a better understanding of how these factors may affect MCV level in association with CVD and CAD deaths.

In conclusion, the results of this study demonstrated that elevated MCV dose-dependently increased the mortality rates attributed to CVD and CAD. This finding supports the use of MCV as a new biomarker for the prediction of risk of death due to CVD and CAD, but a larger study is required to elucidate the specific influences on IS and IHD.

## Methods

### Study population and design

The study population was gathered from an outreach, integrated screening program in Keelung, known as the KCIS program, which was launched in September 1999 to evaluate characteristics related to five types of cancer (breast, colorectal, cervical, liver, and oral) and three chronic diseases (diabetes, hypertension, and hyperlipidemia)^31^. The KCIS program combined health evaluations for these diseases with a routine check of biomarkers and a physical examination. Residents aged 20 years and older based on a population-based household registry were invited to participate. This outreach program still exists and serves approximately 20,000 residents annually. The coverage rate of the KCIS program exceeds 65%.

The KCIS database was utilized to conduct a longitudinal study to investigate the effect of baseline MCV on mortality from CVD or CAD. To ensure adequate induction time for the occurrence of stroke or CAD, the study subjects (limited to enrollees aged 40 years or older) were analyzed between 2000 and 2009 and were followed until the end of 2010. The median follow-up time was 6.21 years with minimum and maximum follow-up times of 0.03 and 8.75 years, respectively. The distribution of time for the 25^th^ and 75^th^ percentiles was 4.31 and 7.51 years, respectively.

### Data collection

Those who participated in the KCIS outreach screening program were given a structured questionnaire by well-trained public health nurses to collect demographic features, anthropometric measurements, lifestyle factors, biomarkers, and self-reported family histories of cancer and chronic diseases. Information on alcohol consumption and cigarette smoking was also obtained, including age at commencement, current status (never, quit, current), duration of cessation, frequency and length of time. Further details are described elsewhere^32,33^. Both systolic blood pressure (SBP) and diastolic blood pressure (DBP) were measured twice with intervals of more than 20 minutes. The lower of the two values was used in this study. The procedure for measuring blood pressure was also described previously^33^. Exercise was defined as non-occupational physical activity and was also collected as a dichotomous variable (Yes/No) from the participants using the question, “Do you perform leisure-time exercise at least once regularly per week with a duration of at least 30 mins each time?” Those who answered “Yes” were defined as frequent exercisers, and the others were defined as infrequent exercises. Moreover, as the aim of this study was to evaluate the effect of changes in MCV, those who attended the program two or more times during the study period were focused on.

### MCV measurement and other biochemical assays

A whole blood examination, including WBC, RBC, hemoglobin, platelet, and MCV measurements, was performed. According to standard laboratory measurements of MCV, those who had MCV >99 fL and <80 fL were classified as high and low MCV levels, respectively. In addition to two extreme criteria, the middle values were categorized as 80–94.9 fL and 95–98.9 fL. The crude mortality rates of CVD/CAD were 1.44, 1.39, 1.92, and 3.99 per 1000 for <80, 80–94.9, 95–98.9, and >99 fL (see Table 1). Considering the similar mortality rate and the too few samples for more refined categories, the <80 and 80–94.9 groups were combined into one group. To consider those who might have an increased potential death rate, the subjects with MCV < 60 fL were excluded from additional analyses. Anemia was defined as hemoglobin <13 for males and hemoglobin <12 for females. The biomarkers analyzed included 8-hour fasting plasma glucose, high-density lipoprotein (HDL), and triglycerides (TG). To determine whether changes in MCV affect mortality, the baseline MCV was subtracted from the last MCV and used as the continuous difference in MCV. Dichotomous changes in MCV were categorized using the cutoff of the mean difference.

All biochemical biomarkers were analyzed by a central laboratory certified by the National Association of Quality Assurance and Control. Blood samples were collected, and questionnaires were completed during the outreach screening. This study was reviewed by the Ethics Committee of Chang Gung Memorial Hospital and was approved by the Institutional Review Board with Issued Numbers 103-4784B and 104-0263C.

### Definition of specific causes of death

The International Classification of Disease (ICD)-9 was used for National Mortality Registry Database coding, but the ICD-10 has been used since 2009. Specific causes of death were identified with the following codes: cerebral IS: 433, 434, 436, 437; CHS: 430, 431, 432; hypertensive disease: 401, 402, 403, 404, 405; IHD: 410, 411, 412, 413, 414; heart failure: 428; pulmonary heart disease and pulmonary circulation disease: 415, 416, 417; disease of arteries, arterioles, and capillaries: 440, 441, 442 (other aneurysm), 443, 444; other venous embolism and thrombosis: 453; late effect of CVD: 438. The ICD-10 was also utilized in the same manner as the ICD-9^34^. The detailed categories used in this study from the ICD-9 are provided in the supplemental document.

### Definition of metabolic syndrome

The first screening results, defined as the baseline, included biochemical markers, lifestyle factors, previous disease history, and anthropometric data. Metabolic syndrome was defined according to the U.S. National Cholesterol Education Program Adult Treatment Panel III (NCEP-ATP III)^35^, but central obesity was redefined as a waist circumference ≥90 cm in men and ≥80 cm in women based on the Asia-Pacific WHO conference. A TG level ≥150 mg/dL was classified as abnormal. Abnormal HDL was defined as lower than 40 mg/dL for males and as lower than 50 mg/dL for females. Those who had a history of hypertension, a SBP ≥ 130 mmHg or a DBP ≥ 85 mmHg were defined as having abnormal BP. A fasting plasma glucose level ≥ 100 mg/dL or a history of diabetes mellitus (DM) was classified as abnormal.

### Statistical methods

The results obtained at the first screening, including biochemical and anthropometric data, were utilized as the baseline measurements in this study. Causes and dates of death were obtained from the Taiwan National Mortality Registry Database. Time-to-death analysis was conducted to calculate the individual follow-up person-year value from the first date of screening entry to the date of death. Those who were still alive at the end of 2010 or had died from other causes of death (whichever came first) were treated as censored cases. The cumulative mortality rate of specific causes of death at each half-year was estimated using an actual life-table method according to different MCV levels. To check the proportional hazards assumption, a graphical method was used to plot log(−log(survival probability)) against log(time) to check whether subgroups paralleled each other; second, a time-dependent Cox model was applied to check whether the MCV level interacts with time. A Cox proportional hazards regression model was constructed to estimate the hazard ratios (HRs) for different MCV levels with regard to specific causes of death and to obtain the adjusted HR (aHR) after adjusting for potential confounding factors. The parsimony model for each Cox proportional hazards regression was used with a p-value entry level of less than 0.1. The significance level was set to 5% for all statistical tests. Statistical analysis was performed using SAS, version 9.4 (SAS Institute Inc., Cary, North Carolina, USA).

### Statement of informed consent

Informed consent was obtained from all subjects for inclusion in this study, which was governed by the Health Bureau. This study was reviewed by the Ethics Committee of Chang Gung Memorial Hospital and was approved by the Institutional Review Board with Issued Numbers 103-4784B and 104-0263C.

### Statement of human and animal right

All procedures followed were in accordance with the ethical standards of the responsible committee on human experimentation (institutional and national) and with the Helsinki Declaration of 1975, as revised in 2008.

## Electronic supplementary material

Supplemental file
