# Supplementary material for: Gradient Relationship between Increased Mean Corpuscular Volume and Mortality Associated with Cerebral Ischemic Stroke and Ischemic Heart Disease: A Longitudinal Study on 66,294 Taiwanese
Source: Sci Rep. 2018 Nov 8;8:16517. doi: 10.1038/s41598-018-34403-w (PMC6224537; doi:10.1038/s41598-018-34403-w)
Supplement: Supplementary file 1 — Supplemental file [file 41598_2018_34403_MOESM1_ESM.pdf]

Gradient Relationship between Increased Mean Corpuscular Volume and Mortality Associated with Cerebral Ischemic Stroke and Ischemic Heart Disease: A Longitudinal Study on 66,294 Taiwanese

Tzy-Haw Wu, MD[1], Jean Ching-Yuan Fann, PhD[2], Sam Li-Sheng Chen, PhD[3], Amy Ming-Fang Yen, PhD[3], Chiung-Jung Wen, MD & PhD[4,5], Yun-Ru Lu, MD & PhD[6], Hsiu-Hsi Chen, PhD[7], Sherry Yueh-Hsia Chiu, PhD[8,9], Horng-Huei Liou, MD & PhD[10]

1. Department of Internal Medicine, National Taiwan University Hospital, Taipei, Taiwan
2. Department of Health Industry Management, College of Healthcare Management, Kainan University, Taoyuan, Taiwan
3. School of Oral Hygiene, College of Oral Medicine, Taipei Medical University, Taipei, Taiwan
4. Department of Geriatrics and Gerontology, National Taiwan University Hospital, Taipei, Taiwan
5. Department of Family Medicine, National Taiwan University Hospital, Taipei, Taiwan
6. Department of Neurology, China Medical University Hospital, Taipei, Taiwan
7. Graduate Institute of Epidemiology and Preventive Medicine, College of Public Health, National Taiwan University, Taipei, Taiwan
8. Department of Health Care Management and Healthy Aging Research Center, Chang Gung University, Tao-Yuan, Taiwan
9. Division of Hepatogastroenterology, Department of Internal Medicine, Kaohsiung Chang Gung Memorial Hospital, Kaohsiung, Taiwan
10. Department of Neurology and Pharmacology, National Taiwan University Hospital and College of Medicine, National Taiwan University, Taipei, Taiwan

Supplement

Supplemental Figure 1. Distribution of hazard rate of cerebral ischemic stroke (CIS)

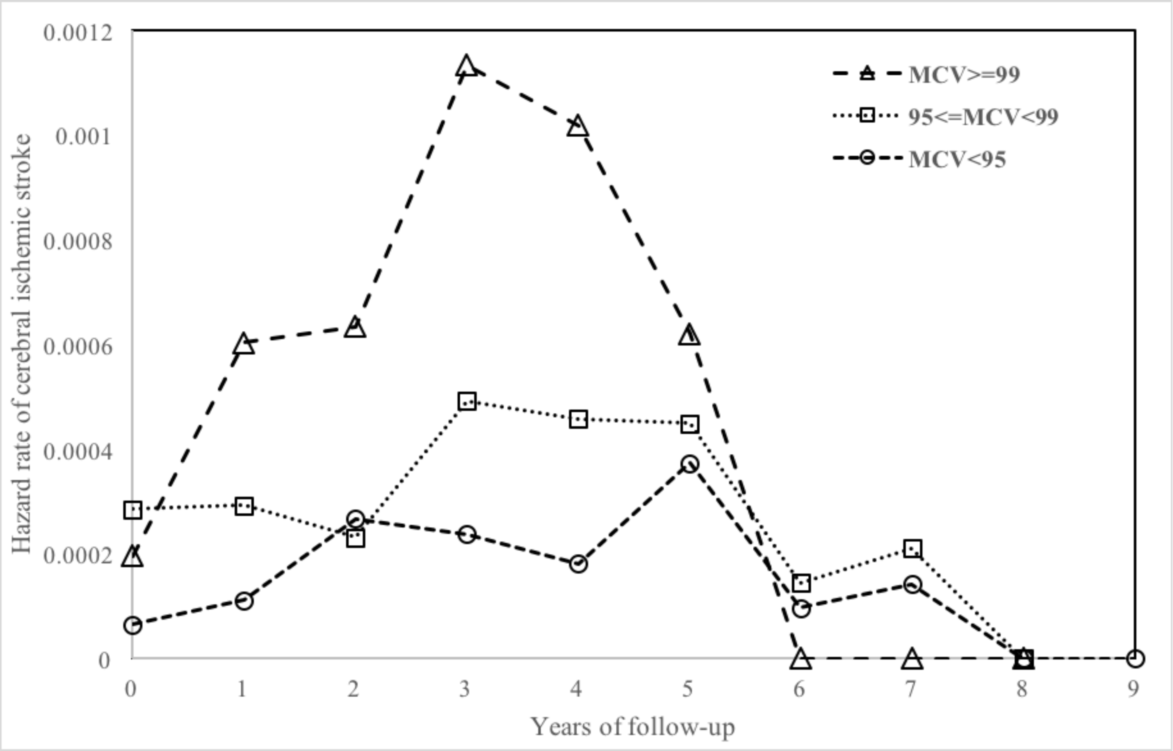

Supplemental Figure 2. Graphic method for proportional hazards assumption checking

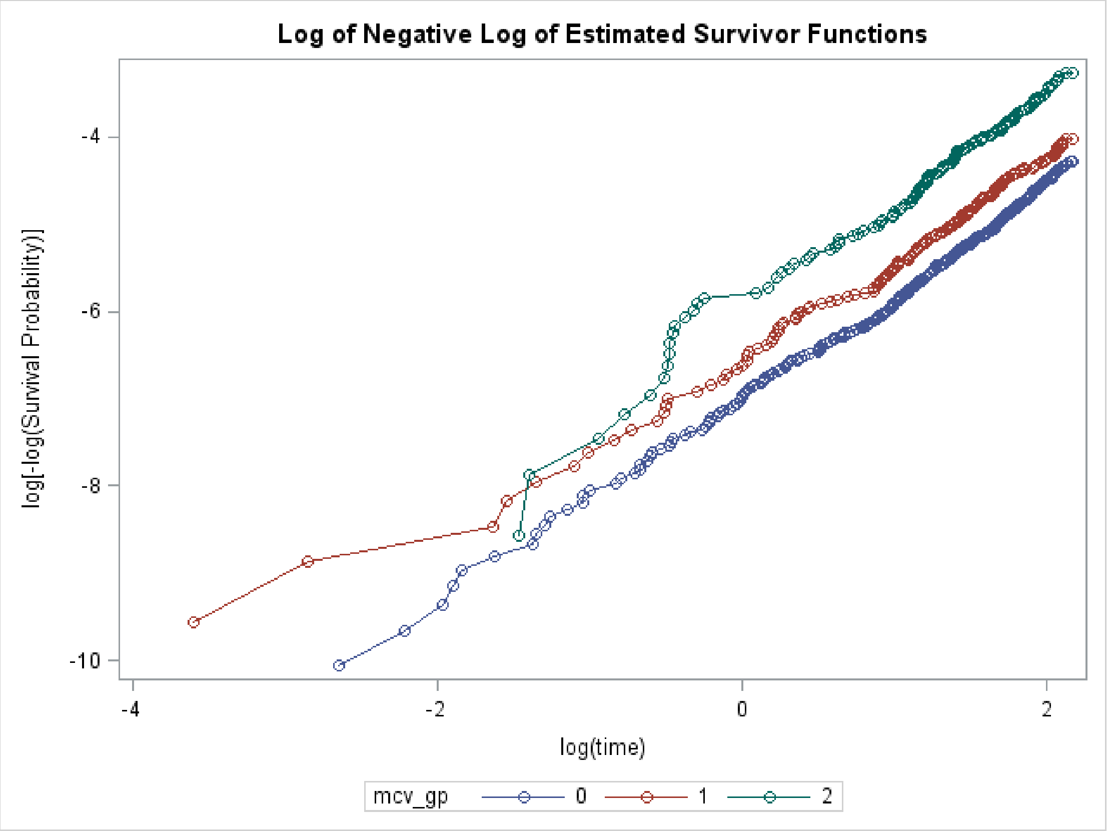

Supplemental Table 1. Crude hazard ratio for risk of specific causes of mortality

Variable	Classification	Cause of death			
		**CVD/CAD related death**	**Cerebral ischemic stroke death**	**Cerebral hemorrhagic stroke death**	**Ischemic heart disease death**
		HR(95%CI)	HR(95%CI)	HR(95%CI)	HR(95%CI)
MCV level	95~98.9 vs. <95	1.36(1.13-1.63)	1.82(1.14-2.90)	1.16(0.72-1.87)	1.34(1.00-1.78)
	>=99 vs. <95	2.84(2.32-3.48)	3.24(1.88-5.56)	1.95(1.09-3.49)	2.98(2.18-4.07)
Age		1.12(1.112-1.13)	1.12(1.10-1.14)	1.09(1.07-1.11)	1.13(1.12-1.14)
Gender	Male vs. female	2.39(2.04-2.79)	2.24(1.49-3.37)	1.55(1.05-2.30)	2.61(2.04-3.33)
Years of education	6-12 vs. >12 yrs	1.70(1.14-2.55)	5.42(0.72-40.75)	2.06(0.72-5.90)	1.23(0.70-2.16)
	<6 vs. >12 yrs	5.30(3.62-7.76)	24.51(3.41-176.28)	5.52(2.01-15.11)	4.11(2.43-6.94)
Waist	Abnormal vs. normal	1.79(1.54-2.09)	1.60(1.07-2.40)	1.28(0.86-1.91)	1.82(1.43-2.31)
Triglyceride	Abnormal vs. normal	1.38(1.18-1.62)	0.99(0.64-1.53)	1.39(0.93-2.08)	1.51(1.18-1.92)
HDL	Abnormal vs. normal	1.55(1.33-1.82)	1.54(1.01-2.34)	1.19(0.78-1.83)	1.73(1.35-2.21)
Blood pressure	Abnormal vs. normal	2.83(2.36-3.40)	3.48(2.08-5.82)	3.25(1.99-5.30)	2.44(1.85-3.21)
FPG	Abnormal vs. normal	2.37(2.03-2.75)	2.87(1.92-4.31)	2.03(1.36-3.01)	2.32(1.83-2.94)
Cigarette smoking	Quit vs. none	1.54(1.17-2.04)	1.51(0.72-3.15)	1.46(0.73-2.92)	1.37(0.86-2.17)
	Current vs. none	1.59(1.34-1.90)	1.43(0.89-2.31)	0.91(0.54-1.54)	1.78(1.37-2.33)
Alcohol drinking	Quit vs. none	2.05(1.53-2.74)	2.43(1.17-5.06)	1.66(0.72-3.80)	2.11(1.34-3.30)
	Current vs. none	0.91(0.74-1.11)	1.00(0.59-1.71)	0.91(0.54-1.54)	0.80(0.58-1.12)
Anemia	Yes vs. no	2.80(2.33-3.36)	2.53(1.53-4.18)	2.67(1.65-4.33)	2.98(2.25-3.94)
WBC	≥6.2 vs. <6.2	1.56(1.33-1.82)	1.49(0.98-2.25)	1.37(0.92-2.05)	1.57(1.23-2.00)
AST/ALT ratio	>1 vs. ≦1	1.87(1.58-2.21)	2.38(1.49, 3.81)	1.78(1.16-2.74)	1.80(1.39-2.33)
eGFR	<60 vs. ≥60	8.24(7.08-9.60)	9.78(6.52-14.66)	5.63(3.76-8.42)	8.06(6.36-10.22)
Seafood intake	≥3 vs. 0-2	0.82(0.71-0.96)	0.90(0.60-1.35)	0.93(0.62-1.39)	0.83(0.65-1.05)
Bean intake	≥3 vs. 0-2	0.72(0.62-0.85)	0.57(0.37-0.87)	0.76(0.51-1.14)	0.74(0.58-0.95)
Milk intake	≥3 vs. 0-2	1.04(0.89-1.22)	1.20(0.79-1.80)	0.97(0.65-1.44)	1.06(0.83-1.35)
Meat intake	≥2 vs. 0-1	0.84(0.69-1.02)	0.76(0.45-1.28)	0.81(0.49-1.34)	0.97(0.73-1.30)
Fruit intake	≥3 vs. 0-2	0.57(0.48-0.68)	0.59(0.38-0.93)	0.83(0.52-1.33)	0.51(0.39-0.66)
Vegetable intake	≥2 vs. 0-1	0.80(0.67-0.96)	0.85(0.54-1.36)	0.63(0.39-1.04)	0.97(0.75-1.27)
Exercise	Regular vs. Irregular	0.84(0.72-0.98)	0.98(0.64-1.49)	0.73(0.49-1.10)	0.88(0.68-1.12)

MCV: Mean corpuscular volume

FPG: fasting plasma glucose; Anemia: male with hemoglobin <13 or female with hemoglobin <12#

Supplemental Table 2. Adjusted hazard ratio for risk of specific causes of mortality

Variable	Classification	**CVD/CAD-related death**	**Cerebral ischemic stroke death**	**Cerebral hemorrhagic stroke death**	**Ischemic heart disease death**
		aHR (95% CI)	aHR (95% CI)	aHR (95% CI)	aHR (95% CI)
MCV level	95-98.9 vs. <95	0.96 (0.80-1.16)	1.32 (0.82-2.12)	0.92(0.56-1.49)	0.91 (0.68-1.23)
	≥99 vs. <95	1.49 (1.21-1.83)	1.74 (1.00-3.02)	1.21(0.67-2.19)	1.49 (1.08-2.06)
Trend test (p-value)		0.0032	0.0491	0.4708	0.0453
Age		1.11 (1.10-1.12)	1.10 (1.07-1.12)	1.08 (1.06-1.10)	1.12 (1.11-1.14)
Gender	Male vs. female	1.61 (1.33-1.95)	1.63 (1.02-2.60)	1.15 (0.77-1.73)	1.69 (1.27-2.25)
Years of education	6-12 vs. >12 yr	1.56 (1.04-2.33)	4.97 (0.66-37.38)	-	-
	<6 vs. >12 yr	1.82 (1.24-2.69)	8.97 (1.24-65.13)	-	-
HDL	Abnormal vs. normal	1.36 (1.15-1.60)	-	-	1.53 (1.19-1.97)
Blood pressure	Abnormal vs. normal	1.39 (1.15-1.67)	1.64 (0.97-2.77)	1.91 (1.15-3.15)	-
FPG	Abnormal vs. normal	1.32 (1.13-1.55)	1.62 (1.08-2.45)	-	1.32 (1.03-1.68)
Cigarette smoking	Quit vs. none	1.10 (0.80-1.50)	-	-	0.91 (0.54-1.53)
	Current vs. none	1.39 (1.14-1.71)	-	-	1.57 (1.16-2.13)
Alcohol drinking	Quit vs. none	1.53 (1.10-2.11)	2.01 (0.94-4.30)	-	1.63 (1.00-2.66)
	Current vs. none	0.97 (0.77-1.22)	1.16 (0.65-2.07)	-	0.86 (0.60-1.24)
Anemia	Yes vs. no	2.12 (1.75-2.56)	2.01 (1.20-3.39)	2.32 (1.41-3.80)	2.14 (1.59-2.87)
WBC	≥6.2 vs. <6.2	1.36 (1.16-1.60)	1.33 (0.87-2.02)	1.33 (0.89-2.00)	1.28 (1.07-1.77)

MCV: Mean corpuscular volume

FPG: fasting plasma glucose; Anemia: males, hemoglobin <13; females, hemoglobin <12

#

Supplemental Table 3. The multivariable analysis using hemoglobin instead of anemia

Variable	Classification	Cause of death			
		**CVD/CAD related death**	**Cerebral ischemic stroke death**	**Cerebral hemorrhagic stroke death**	**Ischemic heart disease death**
		HR(95%CI)	HR(95%CI)	HR(95%CI)	HR(95%CI)
MCV level	95~98.9 vs. <95	0.96(0.80-1.16)	1.34(0.83, 2.15)	0.94(0.58-1.54)	0.91(0.68-1.23)
	>=99 vs. <95	1.48(1.20-1.83)	1.73(0.99, 3.02)	1.23(0.68-2.22)	1.48(1.08-2.04)
Trend test(p-value)		0.0034	0.0474	0.5281	0.0489
Hemoglobin	Continuous	0.82(0.78-0.86)	0.80(0.71-0.92)	0.76(0.68-0.86)	0.82(0.76-0.88)

*multivariable analysis adjusted for same variables in Supplemental Table 2.

Supplemental Table 4. The correlation among hematological variables

Variable	HB	RBC*1000	MCV	WBC
HB	1.0000	0.5772	0.3555	0.1965
		<.0001	<.0001	<.0001
RBC*1000		1.0000	-0.4896	0.1935
			<.0001	<.0001
MCV			1.0000	-0.0065
				0.0946
WBC				1.0000

Supplemental document. ICD9 codes classification for CVD/CAD-related death

Cerebral ischemic stroke (CIS)

433 Occlusion and stenosis of precerebral arteries

434 Occlusion of cerebral arteries

436 Acute, but ill-defined, cerebrovascular disease

437 Other and ill-defined cerebrovascular disease

Cerebral hemorrhagic stroke (CHS)

430 Subarachnoid hemorrhage

431 Intracerebral hemorrhage

432 Other and unspecified intracranial hemorrhage

Hypertensive disease

401 Essential hypertension

402 Hypertensive heart disease

403 Hypertensive chronic kidney disease

404 Hypertensive heart and chronic kidney disease

405 Secondary hypertension

Ischemic heart disease (IHD)

410 Acute myocardial infarction

411 Other acute and subacute forms of ischemic heart disease

412 Old myocardial infarction

413 Angina pectoris

414 Other forms of chronic ischemic heart disease

Heart Failure

428 Heart failure

Pulmonary heart disease and pulmonary circulation disease

415 Acute pulmonary heart disease

416 Chronic pulmonary heart disease

417 Other diseases of pulmonary circulation

Disease of arteries, and capillaries

440 Atherosclerosis

441 Aortic aneurysm and dissection

442 Other aneurysm

443 Other peripheral vascular disease

444 Arterial embolism and thrombosis

Other venous embolism and thrombosis

453 Other venous embolism and thrombosis

Late effect of CVD

438 Late effects of cerebrovascular disease
